# Fecal Microbiome Analysis Distinguishes Bacterial Taxa Biomarkers Associated with Red Fillet Color in Rainbow Trout

**DOI:** 10.3390/microorganisms11112704

**Published:** 2023-11-04

**Authors:** Ridwan O. Ahmed, Ali Ali, Tim Leeds, Mohamed Salem

**Affiliations:** 1 Department of Animal and Avian Sciences, University of Maryland, College Park, MD 20742, USA; rahmed20@umd.edu (R.O.A.); areali@umd.edu (A.A.); 2 United States Department of Agriculture Kearneysville, National Center for Cool and Cold Water Aquaculture, Agricultural Research Service, Kearneysville, WV 25430, USA; tim.leeds@usda.gov

**Keywords:** aquaculture, biomarker, microbiome, pigmentation, carotenoids

## Abstract

The characteristic reddish-pink fillet color of rainbow trout is an important marketing trait. The gastrointestinal microbiome is vital for host health, immunity, and nutrient balance. Host genetics play a crucial role in determining the gut microbiome, and the host–microbiome interaction impacts the host’s phenotypic expression. We hypothesized that fecal microbiota could be used to predict fillet color in rainbow trout. Fish were fed Astaxanthin-supplemented feed for six months, after which 16s rDNA sequencing was used to investigate the fecal microbiome composition in rainbow trout families with reddish-pink fillet coloration (red fillet group, average saturation index = 26.50 ± 2.86) compared to families with pale white fillet color (white fillet group, average saturation index = 21.21 ± 3.53). The linear discriminant analysis effect size (LEFse) tool was used to identify bacterial biomarkers associated with fillet color. The alpha diversity measure shows no difference in the red and white fillet groups. Beta diversity principal component analysis showed clustering of the samples along the white versus red fillet group. The red fillet group has enrichment (LDA score > 1.5) of taxa *Leuconostoc lactis*, *Corynebacterium variabile*, *Jeotgalicoccus halotolerans*, and *Leucobacter chromiireducens*. In contrast, the white fillet group has an enriched presence of *mycoplasma*, *Lachnoclostridium*, and *Oceanobacillus indicireducens*. The enriched bacterial taxa in the red fillet group have probiotic functions and can generate carotenoid pigments. Bacteria taxa enriched in the white fillet group are either commensal, parasitic, or capable of reducing indigo dye. The study identified specific bacterial biomarkers differentially abundant in fish families of divergent fillet color that could be used in genetic selection to improve feed carotenoid retention and reddish-pink fillet color. This work extends our understanding of carotenoid metabolism in rainbow trout through the interaction between gut microbiota and fillet color.

## 1. Introduction

The characteristic reddish-pink fillet color of salmonids is an important quality criterion determining consumers’ purchasing decisions. Carotenoids are organic molecular pigments synthesized by plants, certain bacteria, algae, and fungi [1]. In the natural marine habitat, salmonid fish, including rainbow trout and Atlantic salmon, feed on sea algae and small crustaceans, giving the muscle characteristic pink/reddish coloration. In commercial and farmed aquaculture, synthetic carotenoids, especially astaxanthin, are added as feed additives to provide similar fillet coloration. The astaxanthin deposition rate in the muscle of salmonids is between 1 and 22% [2,3], while up to 30–70% of Astaxanthin supplied in the diet is lost in the feces [4]. This poor utilization of Astaxanthin is troublesome as Astaxanthin is expensive and accounts for up to 25% of the feed cost [5]. Therefore, it is important to investigate the mechanism of carotenoid absorption, metabolism, utilization, and deposition in salmonids, particularly the genes responsible for this metabolism and how they function. It has been suggested that there is a trade-off between the utilization of carotenoids to boost the color of the muscle and its utilization for immune defense and vitamin A production [6,7].

The intestinal microbial community of fish has been demonstrated to be relevant in metabolism within the host [8]. The complex microbial community within the gastrointestinal tract is crucial in the hosts’ health, immunity, and nutrient balance [9,10,11,12]. The fish host–microbial relationship is complex and has been the subject of several studies in fish species in the past [13], including rainbow trout [14,15,16] and other fish, as reviewed by Wang et al. [9]. The fish gastrointestinal microbiome is determined by several factors, including host genetics, environmental factors, and microbial factors, like the adhesion capacity of the microbes [9]. The fish gut microbiota can influence nutrient metabolism through the roles of certain enzyme-producing microbes [17,18,19,20]. The gut microbiome can also protect the fish gastrointestinal tract from infectious agents by assisting in developing and maturing the gut-associated lymphoid tissues (GALT) [9,21,22]. Chapagain et al. [23] proposed that the fecal microbiome is associated with the growth rate in rainbow trout. They reported that amylose degrading and amino acid fermenting bacteria (Clostridium, Leptotrichia, and Peptostreptococcus) are biomarkers of fast growth, while pathogenic bacteria (Corynebacterium and Paeniclostridium) are enriched in slow-growing fish. *Carnobacterium divergens* has been identified as having a probiotic function in Atlantic Salmon [24,25]. Carotenoids can alter the gut microbiota pattern in humans and mice, while certain bacteria can also produce carotenoids within the human gut [26]. Bacteria referred to as carotenogenic bacteria, such as *Firmicutes, Bacteroidetes*, *Actinobacteria, Proteobacteria*, *and Verrucomicrobiota*, can synthesize carotenoids [27,28,29,30]. Therefore, the gut microbiome has emerged as a strong candidate for factors affecting host metabolism, including possible Astaxanthin metabolism.

Astaxanthin supplied in the diet must be absorbed in the intestine, transported to, and then metabolized in the liver before it is deposited in the muscle. We hypothesized that gut microbiota could function as one of the modulators of Astaxanthin absorption/transport and metabolism, which affects fillet color in rainbow trout. Several microorganisms have been proposed to have carotenoid-synthesizing ability via different enzyme pathways [31]. Interestingly, enzymes from different organisms can combine to generate a functional carotenoid biosynthetic pathway in the host, as reviewed by Umeno et al. [32]. The relationship between carotenoids and gut microbiome/carotenoid-producing bacteria in rainbow trout deserves further investigation. Nguyen et al. [33] identified a significant correlation between flesh color and microbiota composition in Atlantic Salmon. Carotenoid-synthesizing bacteria families such as Bacillaceae, *Mycoplasmataceae*, *Pseudomonas*, *Phyllobacteriaceae*, and *Comamonadaceae* were enriched in the fish with more reddish flesh color, while *Pseudoalteromonadaceae*, *Enterobacteriaceae*, *Microbacteriaceae*, and *Vibrionaceae* were in high abundance in the pale individuals. Nguyen et al. [34] identified *Carnobacterium*, a group belonging to the lactic acid bacteria, as strongly related to the flesh color and the evenness of the color between the flesh areas.

Host genetics are crucial in determining the gut microbiome [35]. Host–microbiome interaction and the ultimate impact on the host’s phenotypic expression were previously reviewed in [36]. Buitenhuis et al. [37] found that the proportion of phenotypic variance of milk fatty acid composition explained by rumen microbiome could be up to 0.26–0.42, and including microbiome information in genomic prediction can improve the predictive ability of certain milk fatty acid compositions (C15:0 and C18:3 n-3) by up to 70% (0.22 to 0.38). We hypothesized that the gut microbiota is involved in the metabolism and utilization of Astaxanthin supplied in the diet, thus affecting the fillet color in rainbow trout. In order to develop non-invasive microbial biomarkers that can be used to predict fillet color, we investigated the fecal microbiome composition in rainbow trout families with reddish-pink fillet color compared to families with pale white fillet color. 

## 2. Materials and Methods

### 2.1. Ethical Statement

Husbandry practices and experimental procedures at the facility were approved by the IACUC animal study protocol of the University of Maryland, College Park, protocol number 1593175-6. All methods were carried out in accordance with relevant guidelines and regulations. All methods were carried out in accordance with ARRIVE guidelines [38].

### 2.2. Rainbow Trout Population, Experimental Design, Treatments, and Sampling

The fish used for this study are rainbow trout from a fillet yield genetic selection line developed at the National Center for Cool and Cold Water Aquaculture (NCCCWA). This line started as a growth-selected line in 2002 and underwent five generations of selection for improved growth performance, as described by Leeds et al. [39]. Subsequent generations were selected for muscle yield, as described in Cleveland et al. [40] and Garcia et al. [41]. Fish from the 2020 year class were included in this study and thus represent 3rd-generation families from lines selected for high (ARS-FY-H) or low (ARS-FY-L) fillet yield. The fish (all-female and immature) were received at 322 days post-hatch and randomly allocated to 20 six-foot tanks within the Crane Aquaculture facility of the University of Maryland, College Park. The aquaculture facility uses a recirculating aquaculture system (RAS) with all water quality parameters (water temperature, dissolved oxygen, ammonia concentration) closely monitored and controlled to ensure the fish are in good condition. The water temperature and pH averages 15.0 °C and 7.5, respectively. Dissolved oxygen, ammonia, nitrate and nitrite concentrations are >5 mg/L, ~0.25 mg/L, <2.0 mg/L and <2.0 mg/L, respectively.

The fish were fed an Astaxanthin-supplemented diet (BioTrout 4.0 mm & 6.0 mm, up to 40 ppm Astaxanthin) from Bio-Oregon (Washington, DC, USA) at 3% of their body weight once a day from 322 days post-hatch till harvest. They were taken off feed a day before harvest. During harvest, the fish were euthanized using physical stunning through a blow to the skull with a blunt wooden instrument, immediately followed by exsanguination. They were allowed to undergo rigor mortis on ice for 48 h after harvest and manually processed into trimmed, skinless fillets on the third day. Fecal samples were collected from all fish at two time points per fish (March: age 380 days post-hatch, and June: age 450–485 days post-hatch) and stored in ethanol at −20 °C. The mean body weights were 347.10 g and 694.36 g in March and June sampling time, respectively.

A 7.5 cm × 5 cm skinless raw fillet sample was taken from the right-side fillet at a position beginning about 1.5 cm before the dorsal fin and over the lateral line. All samples were prepared at a uniform thickness of 1 cm to prevent the influence of fillet sample thicknesses on the color measurements. The color was measured on the collected section with the Minolta Chroma Meter CR-200 device (Minolta, Model CR-300; Minolta Camera Co., Osaka, Japan), which gives readings for redness (a*), yellowness (b*), and lightness (L*). Two measurements were taken from the same collected section, and the average value was used.

The saturation index (SI) (a*^2^ + b*^2^)^0.5^ was calculated for all fish, and the average SI value for each family was used to sort the 40 families into “red fillet group” for fish families of high saturation index and “white fillet group” for fish families of low saturation index value. This study used two families from the white (five fish each) group and two from the red fillet (five fish each) group. The saturation index describes the brightness of the color [42].

### 2.3. DNA Extraction

A commercial DNA extraction kit (ZymoBIOMICS*^®^*-96 MagBead DNA Kit, Zymo Research, Irvine, CA, USA) was used to isolate bacterial DNA from the fecal samples (n = 20 samples total from 5 fish each from the red and white fillet groups), following the manufacturer’s instructions.

### 2.4. Library Preparation

The samples were processed and analyzed for microbiome analysis using the targeted sequencing service of Zymo Research, Irvine, CA, USA. The Quick-16STM Plus NGS Library Prep Kit (Zymo Research, Irvine, CA, USA) was used to target the bacterial 16S rRNA gene. The primer set (Quick-16STM Primer Set V3-V4) was designed to amplify the V3-V4 region of the 16S rRNA gene. These primers were custom-designed by Zymo Research to provide the best coverage of the 16S rRNA gene and maintain high sensitivity. The sequencing library was prepared such that PCR reactions were performed in real-time to control cycles and limit the formation of PCR chimera. The final PCR product was quantified using qPCR fluorescence readings and pooled together using equal molarity. The pooled library was cleaned with the Select-a-Size DNA Clean and Concentrator (Zymo Research, Irvine, CA, USA). Library quantification was performed using TapeStation (Agilent Technologies, Santa Clara, CA, USA) and Qubit (Thermo Fisher Scientific, Waltham, WA, USA).

The ZymoBIOMICS^®^ Microbial Community DNA Standard (Zymo Research, Irvine, CA, USA) was used as a positive control with each targeted library preparation. Negative controls (i.e., blank extraction control and blank library preparation control) were used to assess the bioburden level carried out using the wet lab process. Illumina MiSeq with a v3 reagent kit (600 cycles) was used to sequence the final library. The sequencing was performed using a 10% PhiX spike-in.

### 2.5. Bioinformatics and Statistical Analyses

Unique amplicon sequences were identified from raw reads, and chimeric sequences were removed using the Dada2 pipeline [43]. Taxonomy assignment was performed using Uclust from Qiime v.1.9.1 and a 16S rRNA database internally designed and curated as a reference by the Zymo Research Database. QIIME v.1.9.1 was used for composition visualization, alpha-diversity, and beta-diversity analyses [44]. Taxonomic groups with significant abundance among different groups were identified by linear discriminant analysis for effect size (LEfSe), with the time of sample collection as a covariate [45] using default settings. Those default settings were α parameters with pairwise tests set to 0.05 for both class normality and subclass tests, and the threshold on the logarithmic score of linear discriminate analysis was set to 1.5. PCoA plots were performed with internal scripts. For each sample collection time (March and June), we also separately used LEfSe analysis to identify taxonomic groups showing differential abundance between the red and the white fillet group (α_value = 0.05, LDA > 3).

## 3. Results

### 3.1. Mean Saturation Index (S.I) Values between the Red versus White Fillet Group

**Figure 1:**
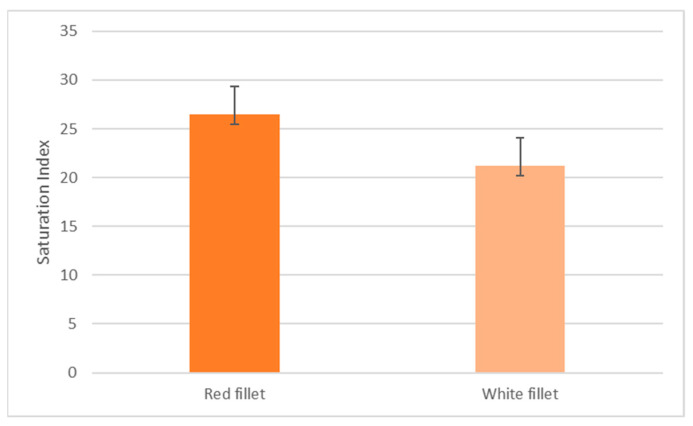
Mean saturation index (S.I) values between the red versus white fillet group.

The mean S.I value of fish in the red and white fillet group is 26.50 ± 2.86 and 21.21 ± 3.53, respectively, as shown in Figure 1. This difference is significant at *p* < 0.05.

### 3.2. Fecal Microbiome Overall Assessment

A total of 9,898,390 16s rDNA raw sequences were obtained, with a range of 403,666 to 628,334 sequences per sample. A total of 17 bacteria phyla were identified: four (*Firmicutes*, *Fusobacteria*, *Proteobacteria*, *Tenericutes*) account for over 97% of the total sequences (Table 1). Bacteria were identified from 290 genera, with 6 genera (*Enterococcus*, *Lactobacillus*, *Peptostreptococcus*, *Romboutsia*, *Cetobacterium*, and *Mycoplasma*) representing 70% of the total sequences. A total of 51 orders, 30 classes, and 100 families were identified.

### 3.3. Fecal Microbiota Composition in Red and White Fillet Fish

#### 3.3.1. Alpha and Beta Diversity

**Figure 2:**
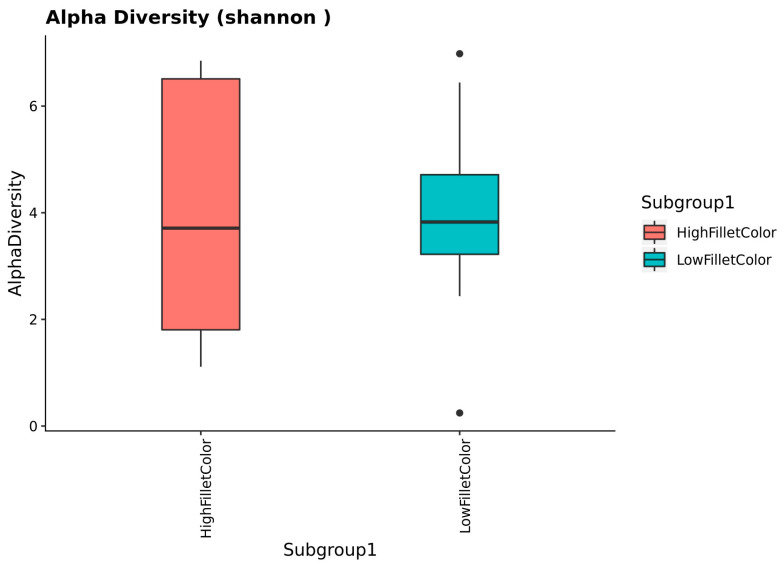
Alpha diversity values between the red (high fillet color) and white (low fillet color) groups.

The alpha diversity assessment using the Wilcoxon rank sum test shows no significant difference between the red and the white fillet group at *p* = 0.05, as shown in Figure 2. The effect of time of sample collection within a group is non-significant.

**Figure 3:**
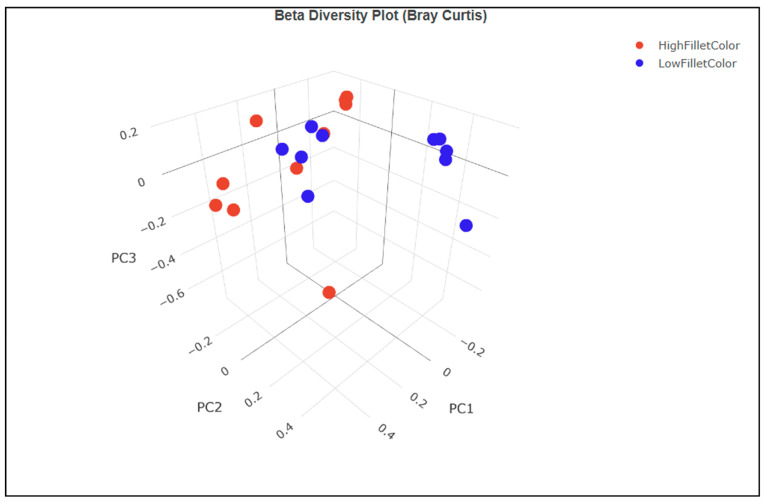
PCA of the beta diversity index showing the red (high fillet color) and white (low fillet color) group clustering.

The principal component analysis (PCA) of the beta diversity index calculated using the Bray–Curtis dissimilarity using unique amplicon sequence variants (ASV) showed clustering of the samples along the white versus red fillet group (Figure 3). Principal components 1 and 2 explain about 33.19% and 22.42% of the total variation in the data, respectively. It shows that the samples from the white fillet group have a more similar microbial community than those from the red fillet group.

#### 3.3.2. Linear Discriminant Analysis for Effect Size

**Figure 4:**
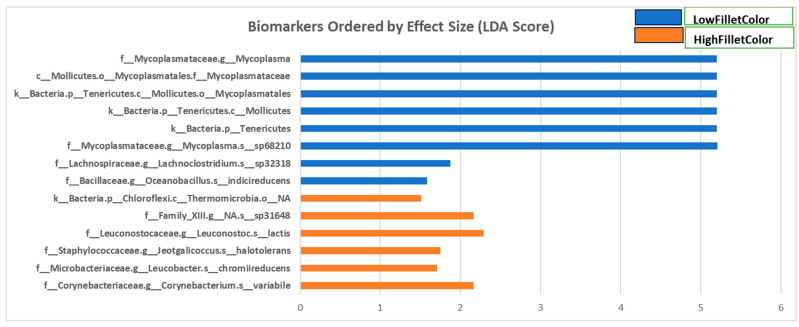
Linear discriminate analysis score of differentially enriched taxa in fecal samples from the red (high) and white (low) fillet group.

Fecal samples were collected at two time points from the fish used in this study: after three and six months (March and June 2021). Figure 4 and Supplementary Table S1 show the phyla, classes, orders, families, and genera with a linear discriminate analysis score greater than 1.5 and a *p*-value less than 0.05 between the two groups when all samples are combined for microbiome composition analyses.

Fecal samples from the red fillet group had more taxa enriched of phyla Actinobacteria and Firmicutes and classes Actinobacteria, Clostridia, and Bacilli. The genera enriched in this group include *Leucobacter*, *Jeotgalicoccus*, *Corynebacterium*, and *Leuconostoc*. In contrast, fecal samples from the white fillet fish had more bacterial taxa enriched from phyla Tenericutes and Firmicutes and classes Mollicutes bacilli and clostridia. The genera enriched in this group are *Lachnoclostridium*, *Oceanobacillus*, and *Mycoplasma*.

In analyzing March and June sampling separately, the LEfSE analysis for the March samples revealed the same differentially enriched taxonomic groups as those observed in the combined analysis (Supplementary Table S1). However, when June fecal samples were analyzed separately, only genera *Mycoplasma* and *Terrisporobacter* were enriched in the white fillet group (Supplementary Table S1).

## 4. Discussion

In this study, we investigated the gut microbiome composition in rainbow trout families with reddish-pink fillet (red fillet group) coloration compared to families with pale white fillet (white fillet group) color to identify bacterial biomarkers associated with fillet color.

There was a significant difference between the color value (saturation index) between the white and the red fillet group. This difference indicates that the red fillet group had more of the desired reddish/pink fillet coloration, and the red group can retain more of the supplemented feed pigment Astaxanthin into the fish’s muscular tissue.

The alpha diversity measure describes the number of bacteria taxa in each sample. There was no significant difference in the alpha diversity measure between the red and the white fillet group. On the other hand, the beta diversity PCA plot showed a separation in the bacteria taxa composition between the red and the white fillet group.

The LEFSe tool was used to identify candidate biomarkers for fillet color in rainbow trout. As discussed below, we found specific taxa indicators of the fillet color groups.

### 4.1. Bacterial Taxa Enriched in the Red Fillet Fish

The taxa *Leuconostoc lactis*, *Corynebacterium variabile*, *Jeotgalicoccus halotolerans*, and *Leucobacter chromiireducens * were significantly more abundant in the red fillet group. 

*Leuconostoc lactis* is a Gram-positive, facultative anerobic lactic acid bacterium. It has been reported to have probiotic and prebiotic effects in the intestinal tract of fish [46,47]. It shows a better adaptive and colonization strategy in the intestine of black porgy fish due to its tolerance to a wide range of pH, bile, trypsin, and pepsin [47]. These properties might be favorable to the host’s health and contribute to improving the fillet quality of the red fillet group fish. Nguyen et al. [34] identified *Carnobacterium * as strongly associated with reddish-pink fillet color in Atlantic salmon. *Carnobacterium* is a bacteria genus belonging to the lactic acid bacteria group, just as *Leuconostoc lactis*, identified as a marker of the red-fillet group in this study. They produce lactic acid as a metabolic end-product of carbohydrate fermentation. Members of the lactic acid bacteria group may be beneficial to the production of reddish-pink fillet. The lactic acid bacteria contribute to the host’s health by acting as probiotics and protecting against diseases [48]. Feeding *Carnobacterium * to rainbow trout increased their survival rate during infection challenge trials [49]. *Corynebacterium variabile* can ferment lactic acid.

The bacteria genus *Corynebacterium*, enriched in the red fillet group, includes a diverse group of non-pathogenic species [50]. *Corynebacterium variabile* can metabolize lactate and has been shown to contribute to the development of flavor and textural properties of cheese during the ripening process [51]. *Corynebacterium variabile,* through the production of pigments, has been suggested as one of the surface bacteria on cheese that contributes to the color development and intensity of the Irish red-smear cheese [52]. The potential of carotenoid production from other bacteria of the genus *Corynebacterium* has been previously reported [53,54,55,56]. They mostly use dimethylallyl pyrophosphate as a precursor in the methylerythritol phosphate pathway to generate carotenoids [54]. The enrichment of *Corynebacterium* in the red fillet group may enhance the red/pink fillet coloration by producing pigments that encourage color development. 

*Jeotgalicoccus halotolerans* is a Gram-positive, anaerobic bacterium first isolated from the Korean fermented seafood jeotgal [57]. It is enriched in the red fillet group. This bacterium is reported to be both halophilic and halotolerant; that is, it can survive in the absence of salt and high salt concentrations. Halophilic bacteria have been reported in the literature as capable of producing carotenoid pigments [58,59,60,61]. The carotenoids produced by this group of bacteria are essential for survival in environments where carotenoids can play a role in membrane stabilization and protection against reactive oxygen species [62]. *Jeotgalicoccus halotolerans* belongs to the order Bacillales. Being a halophilic bacterium, *Jeotgalicoccus halotolerans* may be capable of producing carotenoid pigments that support reddish-pink fillet coloration.

*Leucobacter chromiireducens* is a bacterium first isolated from a chromium-contaminated environment, showing that it is resistant to chromate stress [63]. It is classified as a heavy-metal degrader and possesses chromium reduction ability, which can be used to reduce chromium contamination [64]. The bacteria were enriched in the gut microbiome of fish reared in a polluted river, functioning as a chromium degrader [65]. One mechanism by which *Leucobacter* bacteria respond to chromate stress is to increase the cellular production of carotenoids [66]. Carotenoid production can reduce the concentration of reactive oxygen species and increase cell membrane stability [66,67]. This bacterium’s carotenoid production mechanism might contribute to the red fillet by increasing muscle carotenoid content. 

Evidence from the literature has shown that including certain microorganisms in the diet can improve meat coloration. Skalli et al. [68] showed that the inclusion of microalgae *Scenedesmus sp.* in the diet of rainbow trout significantly improved the fillet yellowness, color saturation, and hue angle. Similarly, the inclusion of the microalgae *Scenedesmus almeriensis,* which contains carotenoid pigments, improves the color intensity and brightness of rainbow trout fillets [69]. *Scenedesmus almeriensis* is rich in lutein, a xanthophyll of yellow coloration, which confers the microalgae the ability to affect skin and fillet color when it is included in the diet of fish [70]. Rainbow trout fed with red algae *Porphyra dioica* meal had a dark orange pigmentation of the fillet compared to the whitish color of the control fish [71]. *Spirulina platensis* supplementation in the diet of rainbow trout significantly improved the carotenoid concentration in the skin and fillet [72]. Similar trends have been reported in other species. Zheng et al. [73] showed that supplementation of the probiotic *Enterococcus faecium* was associated with redder pectoralis muscle in broiler chicken. The inclusion of probiotics (*Bacillus subtilis* endospore and *Clostridium butyricum* endospore complex) in the diet also resulted in darker and redder pig meat [74]. Zhang et al. [75] reported that subjecting the Black Tibetan sheep to different feeding regimes can affect the meat color by altering the abundance of rumen bacteria. The sheep group on pasture grazing in an indoor feeding condition had significantly higher redness (a*) and yellowness (b*) values in their longissimus lumborum muscle and higher abundance of *Lactobacillus*, *Prevotella 1* and *Rikenellaceae RC9 gut* groups in the rumen compared to those fed a concentrate to roughage ratio of 7:3. 

Overall, evidence from this study corroborates the findings from other studies that certain microbiome taxa may support skin/muscle pigmentation and fillet quality through the production of carotenoid pigments and having a probiotic effect within the host. 

### 4.2. Bacterial Taxa Enriched in the White Fillet Fish

*Mycoplasma* (g), *Lachnoclostridium* (g), and *Oceanobacillus indicireducens* are associated with the white fillet color. The bacteria of class mollicutes is the most enriched in the white fillet group. Mycoplasmas (class mollicutes) are small and widespread in humans, plants, animals, and insects. Most of them live as commensals in the host, while others are parasitic, causing chronic infection [76]. Another bacterium enriched in the white fillet group is *Oceanobacillus indicireducens*. It is a facultatively alkaliphilic strain capable of reducing indigo dye extract from the plant *Indigofera tinctoria* through fermentation [77]. Indigo is used to color textiles, and its reduced state is necessary to facilitate solubility in water [78]. *Lachnoclostridium* is a Gram-positive, obligate anaerobic, spore-forming bacterial genus under the class Clostridia [79]. Species under this genus have been identified as markers of several diseases and metabolic conditions in human, such as colorectal adenoma [80], atherosclerosis [81], and serum circulating acetate levels [82]. Two of the three bacteria taxa enriched in the white fillet group have been shown to be pathogenic and may negatively affect the fish and hinder its ability to utilize nutrients and carotenoid for deposition in the muscle.

Similar bacterial taxa as those discussed above were differentially enriched in the March sampling group when analyzed as a stand-alone. However, *Mycoplasma* (genus) and *Terrisporobacter* (genus), found in the white fillet group, were the only enriched taxa when the June samples were analyzed alone. *Terrisporobacter* is an anaerobic acetogenic and pathogenic bacterium capable of degrading carbon sources like xylose and cellobiose [83,84,85]. Differences in bacteria composition were observed in human studies due to differences in fecal sample collection time [86,87]. The result from this study suggests that sample collection time may influence the abundance and composition of bacteria taxa in the gut of rainbow trout.

## 5. Conclusions

The gut microbiome can contribute to phenotypic variation in the host animal. Thus, this study aimed to understand the difference in microbiome composition between two rainbow trout groups with divergent fillet colors. We identified bacteria taxa differentially enriched in white and red-fillet rainbow trout genetic families. The bacteria taxa identified have diverse metabolic pathways that may affect host physiology and fillet color and thus may serve as biomarkers for fillet color. The bacterial taxa community enriched in the red fillet group has probiotic functions and can generate carotenoid pigments, while Mycoplasma, a bacteria taxon that can be pathogenic, is enriched in the white fillet group. This study extends our understanding of the relationship between gut microbiome and fillet color in rainbow trout. The differential abundance of these bacteria taxa could be incorporated into genomic prediction models to accelerate the genetic improvement of fillet color in rainbow trout.

## Figures and Tables

**Table 1:** The percentage taxa abundance of the major phyla and genera identified in the fecal samples.

Phyla	Red Fillet Group	White Fillet Group	Total
*Firmicutes*	22.73	23.73	46.46
*Fusobacteria*	16.31	6.52	22.83
*Proteobacteria*	2.41	2.71	5.12
*Tenericutes*	7.58	15.10	22.68
Others	1.10	1.81	2.91
**Genera**			
*Enterococcus*	4.81	0.35	5.17
*Lactobacillus*	2.71	3.61	6.32
*Peptostreptococcus*	1.71	6.32	8.02
*Romboutsia*	4.86	0.40	5.27
*Cetobacterium*	16.30	6.52	22.82
*Mycoplasma*	7.57	15.10	22.67
Others	12.19	17.55	29.74

## Data Availability

All datasets generated for this study are included in the manuscript and/or the Additional Files. All raw sequence data generated in this study have been deposited in NCBI under BioProject accession number PRJNA974903, https://www.ncbi.nlm.nih.gov/bioproject/PRJNA974903. Registration date: 21 May 2023. Accessed on 3 November 2023.

## Supplementary Materials

The following supporting information can be downloaded at: https://www.mdpi.com/article/10.3390/microorganisms11112704/s1. Table S1: Bacteria biomarkers identified by LEfSE for the March, June and combined March and June analysis with their effect sizes.

## Abbreviations

ASV: amplicon sequence variants, SI: Saturation Index, LEfSe: linear discriminant analysis for effect size, NCCCWA: National Center for Cool and Cold-water Aquaculture.
